# Supplementary material for: Transcutaneous Posterior Tibial Nerve Stimulation: An Adjuvant Treatment for Intractable Constipation in Children
Source: Biomedicines. 2024 Jan 12;12(1):164. doi: 10.3390/biomedicines12010164 (PMC10813187; doi:10.3390/biomedicines12010164)
Supplement: Supplementary file 1 [file biomedicines-12-00164-s001.zip › Supplementary Material.pdf]

Supplementary Material S1

Rego et al. Transcutaneous posterior tibial nerve stimulation: an adjuvant treatment for intractable constipation in children

PROTOCOL NUMBER: ______

CURRENT CLINICAL STATUS

1. How many times do you defecate per day?_____ How many times per week? _____

2. Do you lose stool in underwear without realizing it? ○ Yes ○ No

If yes, how many times a day? ____________ How many times per week?________

3. Is this disrupting your quality of life? ○ Yes ○ No

4. Do you need to wear diapers or underwear protection? ○ Yes ○ No

5. Do you take any medication for bowel habits? ○ Yes ○ No

If yes, what? ____________________________________

6. Do you make use of any kind of special diet? ○ Yes ○ No

If yes, what? ______________

7. Have you had abdominal pain? ○ Yes ○ No

If yes, how many times per week? ____ What is the intensity of the pain (0 to 5)______

8. Have you had straining and pain to pass stool? ○ Yes ○ No

If yes, how many times per week? ____

9. Have you had bleeding during bowel movements? ○ Yes ○ No

If yes, how many times per week? ____

10. Have you had bowel movements of large fecal masses that clog the toilet?

○ Yes ○No If yes, how often? _________

11. Have you had rashes or dermatitis in the perianal region? ○ Yes ○ No

12. Have you had episodes of fecal retention requiring enemas? ○ Yes ○ No

If yes, how often? _________

13. Do you take any medication regularly? ○ Yes ○ No

If yes, what? (which are?)_________________

14. Do you have any other health problems that have required medical attention? ○ Yes ○ No If yes, what?____

Supplementary Material S2

Rego et al. Transcutaneous posterior tibial nerve stimulation: an adjuvant treatment for intractable constipation in children

modified Bristol Stool Form Scale for Children – m-BSFS-C

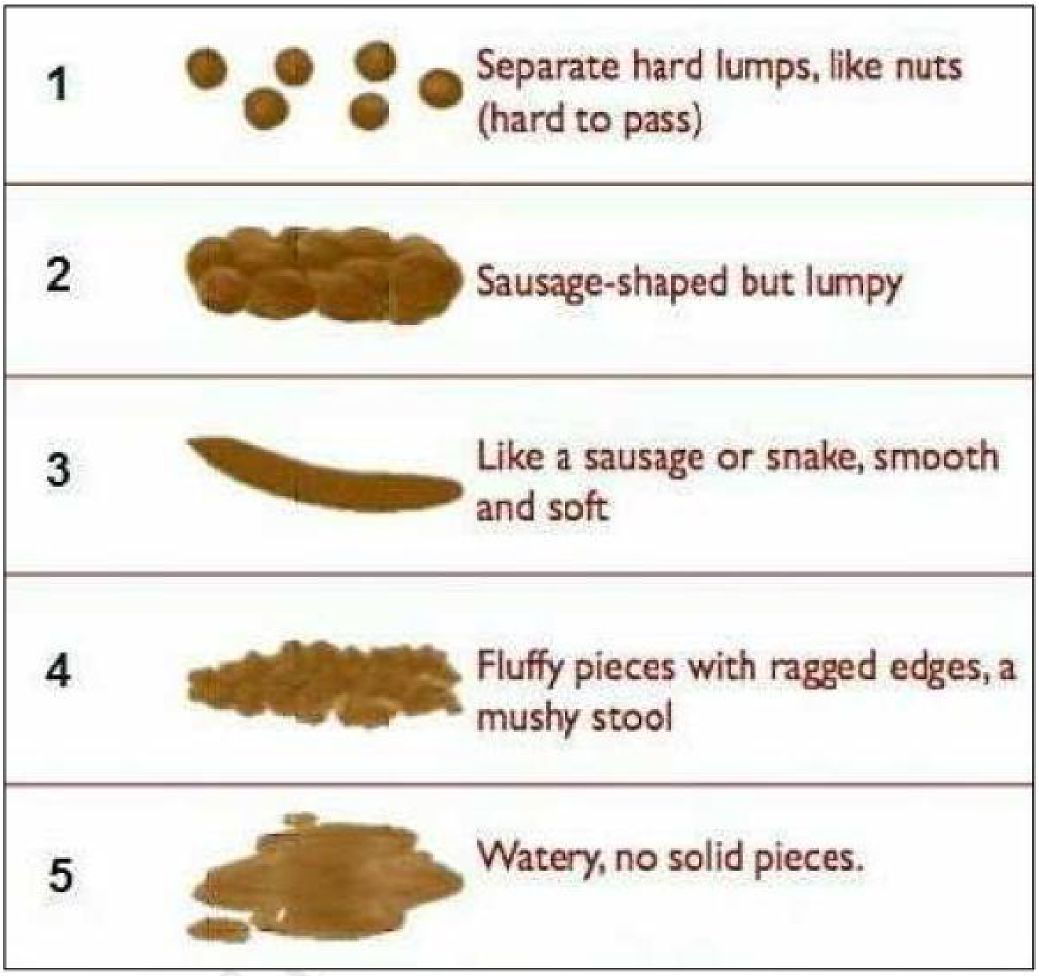

- Chumpitazi BP, Lane MM, Czyzewski DI, Weidler EM, Swank PR, Shulman RJ. Creation and initial evaluation of a stool form scale for children. J Pediatr. 2010;157: 594-597.
- Lane MM, Czyzewski DI, Chumpitazi BP, Shulman RJ. Reliability and validity of a modified Bristol Stool Form Scale for children. J Pediatr. 2011;159:437-441.
- Jozala DR, Oliveira ISF, Ortolan EVP, et al. Brazilian Portuguese translation, cross-cultural adaptation and reproducibility assessment of the modified Bristol Stool Form Scale for children. J Pediatr (Rio J). 2018 Mar 15. pii: S0021-7557(17)31151-8.

Supplementary Material S3

Rego et al. Transcutaneous posterior tibial nerve stimulation: an adjuvant treatment for intractable constipation in children

Bowel Function Score

Factor	Score Given
Ability to hold back defecation	
Always	3
Problems <1/week	2
Weekly problems	1
No voluntary control	0
Feels the urge to defecate	
Always	3
Most of the time	2
Uncertain	1
Absent	0
Frequency of defecation	
Every other day—twice a day	2
More often	1
Less often	1
Soiling	
Never	3
Staining <1/week, no change of underwear required	2
Frequent staining/soiling, change of underwear required	1
Daily soiling, requires protective aids	0
Accidents	
Never	3
Less than 1/week	2
Weekly accidents, often requires protective aids	1
Daily, protective aids required day and night	0
Constipation	
No constipation	3
Manageable with diet	2
Manageable with laxatives	1
Manageable with enemas	0
Social problems	
No social problems	3
Sometimes (foul odors)	2
Problems causing restrictions of social life	1
Major social/psychological problems	0

Adapted from

Jarvi K, Laitakari EM, Koivusalo A, Rintala RJ, Pakarinen MP. Bowel function and gastrointestinal quality of life among adults operated for Hirschsprung disease during childhood: a population-based study. Ann Surg. 2010; 252(6):977-981.

Supplementary Material S4

Rego et al. Transcutaneous posterior tibial nerve stimulation: an adjuvant treatment for intractable constipation in children

User agreement<br><br>Special Terms

Mapi Research Trust, a non-for-profit organisation subject to the terms of the French law of 1st July 1901, registered in Carpentras under number 453 979 346, whose business address is 27 rue de la Villette, 69003 Lyon, France, hereafter referred to as "MRT" and the User, as defined herein, (each referred to singularly as a "Party" and/or collectively as the "Parties"), do hereby agree to the following User Agreement Special and General Terms:

Mapi Research Trust
PROVIDE™
27 rue de la Villette
69003 Lyon
France
Phone: +33 (0)4 72 13 66 66

Recitals

The User acknowledges that it is subject to these Special Terms and to the General Terms of the Agreement, which are included in Appendix 1 to these Special Terms and fully incorporated herein by reference. Under the Agreement, the Questionnaire referenced herein is licensed, not sold, to the User by MRT for use only in accordance with the terms and conditions defined herein. MRT reserves all rights not expressly granted to the User.

The Parties, in these Special Terms, intend to detail the special conditions of their partnership.

The Parties intend that all capitalized terms in the Special Terms have the same definitions as those given in article 1 of the General Terms included in Appendix 1.

In this respect, the Parties have agreed as follows:

Article 1. Conditions Specific to the User

Section 1.01 Identification of the User

User Name	Pedro Luiz Toledo de Arruda Lourenção
Legal Form	University/Hospital
Address	Av. Prof. Mário Rubens Guimarães Montenegro, s/n<br>Departamento de Cirurgia e Ortopedia<br>18618687 Botucatu
Country	Brazil
Email address	plourencao@gmail.com
Telephone number	+551438801703

Section 1.02 Identification of the Questionnaire

© Mapi Research Trust. The unauthorized modification and use of any portion of this document is prohibited.

Title	Pediatric Quality of Life Inventory™ (PedsQL™)
Author(s)	Varni JW
Owner	Varni James W, PhD
Copyright	Copyright © 1998 JW Varni, Ph.D. All rights reserved
Original bibliographic references	See Appendix 2

Article 2. Rights to Use

Section 2.01 Context of the Use of the Questionnaire

The User undertakes to only use the Questionnaire in the context of the Study as defined hereafter.

Context of Use	Clinical project or study
Title	Evaluation of the initial impact of the transcutaneous Posterior Tibial Nerve Stimulation in children with intestinal constipation: protocol for an interventional study.
Disease or condition	intestinal constipation
Type of research	Clinical trial
Study/Protocol reference	The Brazilian Registry of Clinical Trials (Rebec) identifier for this study is RBR-344jq8, obtained on march 13, 2018 (Número do UTN: U1111-1207-5487), available at http://www.ensaiosclinicos.gov.br.
Number of patients expected	28
Number of submissions to the questionnaire for each patient	1
Term of clinical follow-up for each patient	Yes
Start	04/2019
End	04/2020
Mode of administration	Paper administration

Section 2.02 Conditions for Use

The User undertakes to use the Questionnaire in accordance with the conditions for use defined hereafter.

(a) Rights transferred
Acting in the Owner's name, MRT transfers the following limited, non-exclusive rights, to the User (the "Limited Rights")

(i) to use the Questionnaire, only as part of the Study; this right is made up exclusively of the right to communicate it to the Beneficiaries only, free of charge, by any means of communication and by any means of remote distribution known or unknown to date, subject to respecting the conditions for use described hereafter; and

(ii) to reproduce the Questionnaire, only as part of the Study; this right is made up exclusively of the right to physically establish the Questionnaire or to have it physically established, on any paper, electronic, analog or digital medium, and in particular documents, articles, studies, observations, publications, websites whether or not protected by restricted access, CD, DVD, CD-ROM, hard disk, USB flash drive, for the Beneficiaries only and subject to respecting the conditions for use described hereafter; and

© Mapi Research Trust. The unauthorized modification and use of any portion of this document is prohibited.

(iii) Should the Questionnaire not already have been translated into the language requested, the User is entitled to translate the Questionnaire or have it translated in this language, subject to informing MRT of the same beforehand by the signature of a Translation Agreement indicating the terms of it and to providing a copy of the translation thus obtained as soon as possible to MRT.

The User acknowledges and accepts that it is not entitled to amend, modify, condense, adapt, reorganise the Questionnaire on any medium whatsoever, in any way whatsoever, even minor, without MRT's prior specific written consent.

(b) Specific conditions for the Owner

The Owner has intended to transfer a part of the copyright on the Questionnaire and/or the Documentation to MRT in order to enable MRT to make it available to the User for the purpose of the Study, subject to the User respecting the following conditions:

User shall not modify, abridge, condense, translate, adapt, recast or transform the Questionnaire in any manner or form, including but not limited to any minor or significant change in wordings or organisation in the Questionnaire, without the prior written agreement of the Owner. If permission is granted, any improvements, modifications, or enhancements to the Questionnaire which may be conceived or developed, including translations and modules, shall become the property of the Owner.

The User therefore undertakes to respect these special terms.

(c) Specific conditions for the Questionnaire

- Use in Individual clinical practice or Research study / project

The User undertakes never to duplicate, transfer or publish the Questionnaire without indicating the Copyright Notice.

In the case of use of an electronic version of the Questionnaire in academic studies, the User undertakes to respect the following special obligations:

- In case of use of an IT Company (e-vendor), User shall check with Mapi Research Trust that IT Company has signed the necessary License Agreement with Mapi Research Trust before developing the electronic version of the Questionnaire
- Not modify the questionnaire (items and response scales, including the response scale numbers from 0-4)
- Cite the reference publications
- Insert the Owner's copyright notice on all pages/screens on which the Questionnaire will be presented and insert the Trademark information: PedsQL™, Copyright © 1998 JW Varni, Ph.D. All rights reserved.
- Mention the following information: "PedsQL™ contact information and permission to use: Mapi Research Trust, Lyon, France – Internet: https://eprovide.mapi-trust.org and www.pedsql.org/index.html "
- Submit the screenshots of all the Pages where the Questionnaire appears to Dr James W. Varni before release for approval and to check that the above-mentioned requirements have been respected.

In the case of use of an electronic version of the Questionnaire in commercial studies / projects, the User undertakes to respect the following special obligations:

User shall:

- In case of use of an IT Company (e-vendor), User shall check with Mapi Research Trust that IT Company has signed the necessary License Agreement with Mapi Research Trust before developing the electronic version of the Questionnaire
- Not modify the questionnaire (items and response scales, including the response scale numbers from 0-4)
- Cite the reference publications
- Insert the Owner's copyright notice on all pages/screens on which the Questionnaire will be presented and insert the Trademark information: PedsQL™, Copyright © 1998 JW Varni, Ph.D. All rights reserved.
- Mention the following information: "PedsQL™ contact information and permission to use: Mapi Research Trust, Lyon, France – Internet: https://eprovide.mapi-trust.org and www.pedsql.org/index.html"

© Mapi Research Trust. The unauthorized modification and use of any portion of this document is prohibited.

- For the first migration of the Questionnaire (generally the original version) into a specific electronic device

  - Review of screenshots:

After implementation of the Questionnaire into the device, the user/IT Company will generate screen captures (screenshots) of the original questionnaire as displayed in the device. These will be reviewed by Mapi to check that they are consistent with the original paper version in terms of presentation, content and completion except for specific instructions related to the electronic administration. Corrections that may be needed will be reported to the user/IT Company. In this case, screenshots after correction will be generated for another round of review by Mapi until all screenshots are approved.

Dr James W. Varni will review all approved screenshots for a final validation.

  - Usability testing:

Usability testing is a methodology which aims to examine whether respondents are able to use a device and associated software as intended. Major issues of concern in usability testing typically include device complexity, navigation and response selection for example.

The objective of this investigation is to ensure that the electronic version of the questionnaire as included in the device meets usability criteria, focusing on functional aspects and respondents' understanding of instructions. Usability testing consists in interviews with patients where patients will complete the electronic version of the Questionnaire on the device and comment on their understanding of the instructions, ease of use and handiness of the device. A Usability testing report presenting results will be produced. If any changes are recommended, these will be implemented by the user/IT Company. If issues raised by respondents are rated as major, the user/IT Company may need to perform additional developments and another round of interviews may be needed.

Dr James W. Varni will review the changes suggested, if any, following the interviews.

The review of screenshots is mandatory. The usability testing is highly recommended by Mapi, however should the User and/or IT Company decide not to perform this step, Mapi Research Trust shall not be held responsible for any consequence and expense associated with this decision which shall remain the User and/or IT Company's sole liability.

The review of screenshots and usability testing, when and if performed, shall be performed exclusively by Mapi and shall be sponsored by the User.

The performance of the review of screenshots and usability testing will result in a certification of the electronic device original version of the Questionnaires by Mapi for future licenses.

- For the migration of other language versions of the Questionnaire on an existing certified specific electronic device

  - Update version

After the electronic device original version of the Questionnaire is fully ready, the Questionnaire's language versions developed for paper administration will be updated to reflect the changes in wording of instructions implemented in the electronic device original version of the questionnaire.

Native speakers of the languages will reflect the changes made to the electronic device original version of the Questionnaire and will provide English equivalents of all changes made for Mapi's quality control.

  - Review of screenshots:

© Mapi Research Trust. The unauthorized modification and use of any portion of this document is prohibited.

After implementation of the Questionnaire into the device, the user/IT Company will generate screen captures (screenshots) of the original questionnaire as displayed in the device. These will be reviewed by Mapi to check that they are consistent with the original paper version in terms of presentation, content and completion except for specific instructions related to the electronic administration. Corrections that may be needed will be reported to the user/IT Company. In this case, screenshots after correction will be generated for another round of review by Mapi until all screenshots are approved.

The update of version and review of screenshots are mandatory. These steps shall be performed exclusively by Mapi and shall be sponsored by the User.

The performance of the update of version and review of screenshots will result in a certification of the electronic device language version of the Questionnaires by Mapi for future licenses.

- Use in a publication:

In the case of a publication, article, study or observation on paper or electronic format of the Questionnaire, the User undertakes to respect the following special obligations:

- not to include any full copy of the Questionnaire, but a version with the indication "sample copy, do not use without permission"
- to indicate the name and copyright notice of the Owner (PedsQL™, Copyright © 1998 JW Varni, Ph.D. All rights reserved)
- to include the reference publications of the Questionnaire
- to indicate the details of MRT for any information on the Questionnaire as follows: "PedsQL™ contact information and permission to use: Mapi Research Trust, Lyon, France. – Internet: https://eprovide.mapi-trust.org and www.pedsql.org "
- to provide MRT, as soon as possible, with a copy of any publication regarding the Questionnaire, for information purposes
- to submit the screenshots of all the Pages where the Questionnaire appears to MRT before release to check that the above-mentioned requirements have been respected.

- Use for dissemination or marketing:

In the case of use in a dissemination/marketing context:

- On a website with unrestricted access:

The publication of a copy of the PedsQL™ on a website with unrestricted access is not permitted.

- On a website with restricted access:

In the case of publication on a website with restricted access, the User may include a version of the Questionnaire that may be amended, subject to this version being protected by a sufficiently secure access to only allow the Beneficiaries to access it.

Article 3. Term

MRT transfers the Limited Rights to use the Questionnaire as from the date of delivery of the Questionnaire to the User and for the whole period of the Study.

Article 4. Beneficiaries

© Mapi Research Trust. The unauthorized modification and use of any portion of this document is prohibited.

The Parties agree that the User may communicate the Questionnaire in accordance with the conditions defined above to the Beneficiaries involved in the Study only, in relation to the Study defined in section 2.01.

Article 5. Territories and Languages

MRT transfers the Limited Rights to use the Questionnaire on the following territories and in the languages indicated in the table below:

Questionnaire	Language
PedsQL™ Generic Core Scales	Portuguese for Brazil

Article 6. Price and Payment Terms

The User undertakes in relation to MRT to pay the price owed in return for the availability of the Questionnaire, according to the prices set out below, depending on the languages requested and the costs of using the Questionnaire, in accordance with the terms and conditions described in section 6.02 of the General Terms included in Appendix 1.

Access to the Questionnaire in non-funded academic research and individual clinical practice is free of charge.

Agreed and acknowledged by

Pedro Luiz Toledo de Arruda Lourenção

21-Jan-2019

Appendix 1 to the Special Terms: User Agreement General Terms

User has read and accepted the Mapi's General Terms of the Agreement, which are available on MRT's website: https://eprovide.mapi-trust.org/user-agreement-general-terms

Appendix 2 to the Special Terms: References

Generic Core Scales:

- Varni JW, et al. The PedsQL™: Measurement Model for the Pediatric Quality of Life Inventory.Medical Care,1999; 37(2):126-139

© Mapi Research Trust. The unauthorized modification and use of any portion of this document is prohibited.

- Varni, J.W., et al. The PedsQL™ 4.0: Reliability and validity of the Pediatric Quality of Life Inventory™ Version 4.0 Generic Core Scales in healthy and patient populations. Medical Care,2001; 39(8): 800-812.

- Varni, J.W., et al. (2002). The PedsQL™ 4.0 Generic Core Scales: Sensitivity, responsiveness, and impact on clinical decision-making.Journal of Behavioral Medicine, 25, 175-193.

- Varni, J.W., et al. (2003). The PedsQL™ 4.0 as a pediatric population health measure: Feasibility, reliability, and validity.Ambulatory Pediatrics, 3, 329-341.

- Chan, K.S., Mangione-Smith, R., Burwinkle, T.M., Rosen, M., & Varni, J.W. (2005). The PedsQL™: Reliability and validity of the Short-Form Generic Core Scales and Asthma Module.Medical Care, 43, 256-265.

- Varni, J.W., & Limbers, C.A. (2009). The PedsQL™ 4.0 Generic Core Scales Young Adult Version: Feasibility, reliability and validity in a university student population.Journal of Health Psychology, 14, 611-622.

Asthma Module:

- Varni, J.W., Burwinkle, T.M., Rapoff, M.A., Kamps, J.L., & Olson, N. The PedsQL™ in pediatric asthma: Reliability and validity of the Pediatric Quality of Life Inventory™ Generic Core Scales and Asthma Module. Journal of Behavioral Medicine, 2004; 27:297-318.

- Chan, K.S., Mangione-Smith, R., Burwinkle, T.M, Rosen, M., & Varni, J.W. (2005). The PedsQL™: Reliability and validity of the Short-Form Generic Core Scales and Asthma Module. Medical Care, 43, 256-265.

Brain Tumor Module:

- Palmer, S.N., Meeske, K.A., Katz, E.R., Burwinke, T.M., & Varni, J.W. (2007). The PedsQL™ Brain Tumor Module: Initial reliability and validity. Pediatric Blood and Cancer, 49, 287-293.

Cancer Module:

- Varni, J.W., Burwinkle, T.M., Katz, E.R., Meeske, K., & Dickinson, P. The PedsQL™ in pediatric cancer: Reliability and validity of the Pediatric Quality of Life Inventory™ Generic Core Scales, Multidimensional Fatigue Scale, and Cancer Module.Cancer, 2002; 94: 2090-2106.

- Robert RS, Paxton RJ, Palla SL, Yang G, Askins MA, Joy SE, Ater JL. Feasibility, reliability, and validity of the pediatric quality of life inventory™ generic core scales, cancer module, and multidimensional fatigue scale in long-term adult survivors of pediatric cancer.Pediatric Blood & Cancer2012;59:703–707.

Cerebral Palsy Module:

© Mapi Research Trust. The unauthorized modification and use of any portion of this document is prohibited.

- Varni JW, Burwinkle TM, Berrin SJ, Sherman SA, Artavia K, Malcarne VL, Chambers HG (2006). The PedsQL™ in Pediatric Cerebral Palsy: Reliability, Validity, and Sensitivity of the Generic Core Scales and Cerebral Palsy Module.Developmental Medicine and Child Neurology, 48: 442-449.

Cardiac Module:

- Uzark, K., Jones, K., Burwinkle, T.M., & Varni, J.W. The Pediatric Quality of Life Inventory™ in children with heart disease.Progress in Pediatric Cardiology, 2003; 18:141-148.

- Uzark, K., Jones, K., Slusher, J., Limbers, C.A., Burwinkle, T.M., & Varni, J.W. (2008). Quality of life in children with heart disease as perceived by children and parents. Pediatrics, 121, e1060-e1067.

Cognitive Functioning Scale:

- McCarthy, M.L., MacKenzie, E.J., Durbin, D.R., Aitken, M.E., Jaffe, K.M., Paidas, C.N. et al. (2005). The Pediatric Quality of Life Inventory: An evaluation of its reliability and validity for children with traumatic brain injury.Archives of Physical Medicine and Rehabilitation, 86, 1901-1909.

- Varni, J.W., Burwinkle, T.M., Katz, E.R., Meeske, K., & Dickinson, P. (2002). The PedsQL™ in pediatric cancer: Reliability and validity of the Pediatric Quality of Life Inventory Generic Core Scales, Multidimensional Fatigue Scale, and Cancer Module.Cancer, 94, 2090-2106.

- Varni, J.W., Limbers, C.A., Sorensen, L.G., Neighbors, K., Martz, K., Bucuvalas, J.C., & Alonso, E.M. (2011). PedsQL™ Cognitive Functioning Scale in pediatric liver transplant recipients: Feasibility, reliability and validity.Quality of Life Research, 20, 913–921.

Diabetes Module:

- Varni, J.W., Curtis, B.H., Abetz, L.N., Lasch, K.E., Piault, E.C., & Zeytoonjian, A.A. (2013). Content validity of the PedsQL™ 3.2 Diabetes Module in newly diagnosed patients with Type 1 Diabetes Mellitus ages 8-45.Quality of Life Research. 22, 2169–2181.

- Varni, J.W., Burwinkle, T.M., Jacobs, J.R., Gottschalk, M., Kaufman, F., & Jones, K.L. The PedsQL™ in Type 1 and Type 2 diabetes: Reliability and validity of the Pediatric Quality of Life Inventory™ Generic Core Scales and Type 1 Diabetes Module.Diabetes Care, 2003; 26: 631-637.

- Nansel, T.R., Weisberg-Benchell, J., Wysocki, T., Laffel, L. & Anderson, B. (2008). Quality of life in children with Type 1 diabetes: A comparison of general and disease-specific measures and support for a unitary diabetes quality of life construct. Diabetic Medicine, 25, 1316-1323.

- Naughton, M.J., Ruggiero, A.M., Lawrence, J.M., Imperatore, G., Klingensmith, G.J. Waitzfelder, B., McKeown, R.E., Standiford, D.A., Liese, A.D., & Loots, B. (2008). Health-related quality of life of children and adolescents with type 1 or type 2 diabetes mellitus: SEARCH for Diabetes In Youth Study.Archives of Pediatrics and Adolescent Medicine, 162, 649-657.

- Hilliard, M.E., Lawrence, J.M., Modi, A.C., Anderson, A., Crume, T., Dolan, L.M., Merchant, A.T., Yi-Frazier, J.P.,

© Mapi Research Trust. The unauthorized modification and use of any portion of this document is prohibited.

& Hood, K.K. (2013). Identification of minimal clinically important difference scores of the Pediatric Quality of Life Inventory in children, adolescents, and young adults with Type 1 and Type 2 diabetes.Diabetes Care, 36, 1891–1897.

Duchenne Muscular Dystrophy Module:

- Uzark, K., King, E., Cripe, L., Spicer, R., Sage, J., Kinnett, K., Wong, B., Pratt, J., & Varni, J.W. (2012). Health-related quality of life in children and adolescents with Duchenne Muscular Dystrophy.Pediatrics, 130, e1559-e1566.

-

End Stage Renal Disease Module:

- Goldstein, S.L., Graham, N., Warady, B.A., Seikaly, M., McDonald, R., Burwinkle, T.M., Limbers, C.A., & Varni, J.W. (2008). Measuring health-related quality of life in children with ESRD: Performance of the Generic and ESRD-Specific Instrument of the Pediatric Quality of Life Inventory™ (PedsQL™). American Journal of Kidney Diseases, 51, 285-297.

Eosinophilic Esophagitis:

- Franciosi, J.P., Hommel, K.A., Bendo, C.B., King, E.C., Collins, M.H., Eby, M.D., Marsolo, K., Abonia, J.P., von Tiehl, K.F., Putnam, P.E., Greenler, A.J., Greenberg, A.B., Bryson, R.A., Davis, C.M., Olive, A.P., Gupta, S.K., Erwin, E.A., Klinnert, M.D., Spergel, J.M., Denham, J.M., Furuta, G.T., Rothenberg, M.E., & Varni, J.W. (2013). PedsQL™ Eosinophilic Esophagitis Module: Feasibility, reliability and validity.Journal of Pediatric Gastroenterology & Nutrition, 57, 57-66.

- Franciosi, J.P., Hommel, K.A., Greenberg, A.B., Debrosse, C.W., Greenler, A.J., Abonia, J.P., Rothenberg, M.E., & Varni, J.W. (2012). Development of the Pediatric Quality of Life Inventory™ Eosinophilic Esophagitis Module items: Qualitative methods.BMC Gastroenterology, 12:135, 1-8.

- Franciosi J.P., Hommel, K.A., Debrosse, C.W., Greenberg, A.B., Greenler, A.J., Abonia, J.P., Rothenberg, M.E., & Varni, J.W. (2012). Quality of life in paediatric eosinophilic oesophagitis: What is important to patients?Child: Care, Health and Development, 38, 477–483.

Family impact Module:

- Varni, J.W., Sherman, S.A., Burwinkle, T.M., Dickinson, P.E., & Dixon, P. (2004). The PedsQL™ Family Impact Module: Preliminary reliability and validity. Health and Quality of Life Outcomes; 2 (55), 1-6.

- Medrano, G.R., Berlin, K.S., & Davies, W.H. (2013). Utility of the PedsQL™ Family Impact Module: Assessing the psychometric properties in a community sample.Quality of Life Research. 22, 2899-2907.

- Jiang, X., Sun, L., Wang, B., Yang, X., Shang, L., & Zhang, Y. (2013). Health-related quality of life among

© Mapi Research Trust. The unauthorized modification and use of any portion of this document is prohibited.

children with recurrent respiratory tract infections in Xi'an, China.PLoS One, 8(2): e56945.

- Mano, K.E., Khan, K.A., Ladwig, R.J., & Weisman, S.J. (2011). The impact of pediatric chronic pain on parents' health-related quality of life and family functioning: Reliability and validity of the PedsQL 4.0 Family Impact Module.Journal of Pediatric Psychology, 36, 517-527.

Gastrointestinal Symptoms Module:

- Varni, J.W., Bendo, C.B., Denham, J., Shulman, R.J., Self, M.M., Neigut, D.A., Nurko S., Patel, A.S, Franciosi, J.P., Saps, M., Verga, B., Smith, A., Yeckes, A., Heinz, N., Langseder, A., Saeed, S., Zacur, G.M., & Pohl, J.F. (in press). PedsQL™ Gastrointestinal Symptoms Module: Feasibility, reliability, and validity.Journal of Pediatric Gastroenterology & Nutrition.

- Varni, J.W., Bendo, C.B., Denham, J., Shulman, R.J., Self, M.M., Neigut, D.A., Nurko, S., Patel, A.S, Franciosi, J.P., Saps, M., Yeckes, A., Langseder, A., Saeed, S., & Pohl, J.F. (in press). PedsQL™ Gastrointestinal Symptoms Scales and Gastrointestinal Worry Scales in pediatric patients with functional and organic gastrointestinal diseases in comparison to healthy controls.Quality of Life Research.

- Varni, J.W., Kay, M.T., Limbers, C.A., Franciosi, J.P., & Pohl, J.F. (2012). PedsQL™ Gastrointestinal Symptoms Module item development: Qualitative methods.Journal of Pediatric Gastroenterology & Nutrition, 54, 664-671.

Gastrointestinal Symptoms Scales:

- Varni, J.W., Bendo, C.B., Denham, J., Shulman, R.J., Self, M.M., Neigut, D.A., Nurko S., Patel, A.S, Franciosi, J.P., Saps, M., Verga, B., Smith, A., Yeckes, A., Heinz, N., Langseder, A., Saeed, S., Zacur, G.M., & Pohl, J.F. (2014). PedsQL™ Gastrointestinal Symptoms Module: Feasibility, reliability, and validity. Journal of Pediatric Gastroenterology & Nutrition,59, 347–355.

- Varni, J.W., Bendo, C.B., Denham, J., Shulman, R.J., Self, M.M., Neigut, D.A., Nurko, S., Patel, A.S, Franciosi, J.P., Saps, M., Yeckes, A., Langseder, A., Saeed, S., & Pohl, J.F. (in press). PedsQL™ Gastrointestinal Symptoms Scales and Gastrointestinal Worry Scales in pediatric patients with functional and organic gastrointestinal diseases in comparison to healthy controls.Quality of Life Research.

- Varni, J.W., Kay, M.T., Limbers, C.A., Franciosi, J.P., & Pohl, J.F. (2012). PedsQL™ Gastrointestinal Symptoms Module item development: Qualitative methods.Journal of Pediatric Gastroenterology & Nutrition, 54, 664-671.

General Well-Being Scale:

- Varni, J.W., Seid, M., & Kurtin, P.S. (1999). Pediatric health-related quality of life measurement technology: A guide for health care decision makes. Journal of Clinical Outcomes Management, 6, 33-40.

- Hallstrand, T.S., Curtis, J.R., Aitken, M.L., & Sullivan, S.D. (2003). Quality of life in adolescents with mild asthma. Pediatric Pulmonology, 36, 536-543.

© Mapi Research Trust. The unauthorized modification and use of any portion of this document is prohibited.

Mapi Research Trust

Healthcare Satisfaction Generic Module:

- Varni, J.W., Burwinkle, T.M., Dickinson, P., Sherman, S.A., Dixon, P., Ervice, J.A., Leyden, P.A. & Sadler, B.L. (2004). Evaluation of the built environment at a Children's Convalescent Hospital: Development of the Pediatric Quality of Life Inventory™ Parent and Staff Satisfaction Measures for pediatric health care facilities. Journal of Developmental and Behavioral Pediatrics, 2004; 25:10-25.

- Li, J., Yuan, L., Wu, Y., Luan, Y., & Hao, Y. (2013). The Chinese version of the Pediatric Quality of Life Inventory™ (PedsQL™) healthcare satisfaction generic module (version 3.0): Psychometric evaluation. Health and Quality of Life Outcomes, 11(1):113.

- de Souza, F.M., Molina, J., Terreri, M.T., Hilário, M.O., & Len, CA. (2012). Reliability of the Pediatric Quality of Life Inventory - Healthcare Satisfaction Generic Module 3.0 version for the assessment of the quality of care of children with chronic diseases. Journal of Pediatrics (Rio J), 88, 54-60.

Health Care Satisfaction Module specific for Hematology/Oncology:

- Varni, J.W., Quiggins, D.J.L., & Ayala, G.X. (2000). Development of the Pediatric Hematology/Oncology Parent Satisfaction survey. Children's Health Care, 29, 243-255.

Infant Scales:

- Varni, J.W., Limbers, C.A., Neighbors, K., Schulz, K., Lieu, J.E.C., Heffer, R.W., Tuzinkiewicz, K., Mangione-Smith, R., Zimmerman, J.J., & Alonso, E.M. (2011). The PedsQL™ Infant Scales: Feasibility, internal consistency reliability and validity in healthy and ill infants.Quality of Life Research, 20, 45-55.

- Grindler, D.J., Blank, S.J., Schulz, K.A., Witsell, D.L., & Lieu, J.E. (2014). Impact of otitis media severity on children's quality of life.Otolaryngology-Head and Neck Surgery, 151, 333–340.

- Bell, N., Kruse, S., Simons, R.K., & Brussoni, M. (2014). A spatial analysis of functional outcomes and quality of life outcomes after pediatric injury.Injury Epidemiology, 1:16, 1-10.

Multidimensional Fatigue Scale:

- Varni, J.W., Burwinkle, T.M., Katz, E.R., Meeske, K., & Dickinson, P. (2002). The PedsQL™ in pediatric cancer: Reliability and validity of the Pediatric Quality of Life Inventory™ Generic Core Scales, Multidimensional Fatigue Scale, and Cancer Module.Cancer, 94, 2090-2106.

- Varni, J. W., Beaujean, A., & Limbers, C. A. (2013). Factorial invariance of pediatric patient self-reported fatigue across age and gender: A multigroup confirmatory factor analysis approach utilizing the PedsQL™ Multidimensional Fatigue Scale.Quality of Life Research, 22, 2581-2594.

- Varni, J.W., Burwinkle, T.M., & Szer, I.S. (2004). The PedsQL™ Multidimensional Fatigue Scale in

© Mapi Research Trust. The unauthorized modification and use of any portion of this document is prohibited.

pediatric rheumatology: Reliability and validity. Journal of Rheumatology; 31, 2494-2500.

- Varni, J.W., & Limbers, C.A. (2008). The PedsQL™ Multidimensional Fatigue Scale in young adults: Feasibility, reliability and validity in a university student population. Quality of Life Research, 17, 105-114.

- Panepinto, J.A., Torres, S., Bendo, C.B., McCavit, T.L., Dinu, B., Sherman-Bien, S., Bemrich-Stolz, C., & Varni, J.W. (2014). PedsQL™ Multidimensional Fatigue Scale in sickle cell disease: Feasibility, reliability and validity.Pediatric Blood & Cancer, 61, 171–177.

Neurofibromatosis Type 1 Module:

- Nutakki, K., Hingtgen, C.M., Monahan, P., Varni, J.W., & Swigonski, N.L. (2013). Development of the adult PedsQL™ Neurofibromatosis Type 1 Module: Initial feasibility, reliability and validity.Health and Quality of Life Outcomes, 11:21, 1-9

Neuromuscular Module:

- Iannaccone, S.T., Hynan, L.S., Morton, A., Buchanan, R., Limbers, C.A., & Varni, J.W. (2009). The PedsQL™ in pediatric patients with Spinal Muscular Atrophy: Feasibility, reliability, and validity of the Pediatric Quality of Life Inventory™ Generic Core Scales and Neuromuscular Module.Neuromuscular Disorders, 19, 805-812.

- Davis, S.E., Hynan, L.S., Limbers, C.A., Andersen, C.M., Greene, M.C., Varni, J.W., & Iannaccone, S.T. (2010). The PedsQL™ in pediatric patients with Duchenne Muscular Dystrophy: Feasibility, reliability, and validity of the Pediatric Quality of Life Inventory™ Neuromuscular Module and Generic Core Scales.Journal of Clinical Neuromuscular Disease, 11, 97-109.

Oral Health Scale:

- Steele, M.M., Steele, R.G., & Varni, J.W. (2009). Reliability and validity of the PedsQL™ Oral Health Scale: Measuring the relationship between child oral health and health-related quality of life.Children's Health Care, 38, 228-224.

Pediatric Pain Coping Inventory™:

- Varni, J.W., Waldron, S.A., Gragg, R.A., Rapoff, M.A., Bernstein, B.H., Lindsley, C.B., & Newcomb, M.D (1996). Development of the Waldron/Varni Pediatric Pain Coping Inventory. Pain, 67, 141-150.

Pediatric Pain Questionnaire:

- Varni, J.W., Thompson, K.L., & Hanson, V. (1987). The Varni/Thompson Pediatric Pain Questionnaire: I. Chronic musculoskeletal pain in juvenile rheumatoid arthritis.Pain, 28, 27-38.

© Mapi Research Trust. The unauthorized modification and use of any portion of this document is prohibited.

Present Functioning Visual Analogue Scales:

- Sherman, S.A., Eisen, S., Burwinkle, T.M., & Varni, J.W. (2006). The PedsQL™ Present Functioning Visual Analogue Scales: Preliminary reliability and validity.Health and Quality of Life Outcomes, 4:75, 1-10.

Sickle Cell Disease Module:

- Panepinto, J.A., Torres, S., Bendo, C.B., McCavit, T.L., Dinu, B., Sherman-Bien, S., Bemrich-Stolz, C., & Varni, J.W. (2013). PedsQL™ Sickle Cell Disease Module: Feasibility, reliability and validity.Pediatric Blood & Cancer, 60, 1338–1344.

- Panepinto, J.A., Torres, S., & Varni. J.W. (2012). Development of the PedsQL™ Sickle Cell Disease Module items: Qualitative methods.Quality of Life Research, 21, 341-357.

Stem Cell Transplant Module:

- Lawitschka, A., Güclü, E.D., Varni, J.W., Putz, M., Wolff, D., Pavletic, S., Greinix, H., Peters, C., & Felder-Puig, R. (2014). Health-related quality of life in pediatric patients after allogeneic SCT: Development of the PedsQL™ Stem Cell Transplant Module and results of a pilot study. Bone Marrow Transplantation, 49, 1093–1097.

Rheumatology Module:

- Varni, J.W., Seid, M., Knight, T.S., Burwinkle, T.M., Brown, J., & Szer, I.S. (2002). The PedsQL™ in pediatric rheumatology: Reliability, validity, and responsiveness of the Pediatric Quality of Life Inventory™ Generic Core Scales and Rheumatology Module.Arthritis and Rheumatism, 2002; 46: 714-725.

Transplant Module:

- Weissberg-Benchell, J., Zielinski, T.E., Rodgers, S., Greenley, R.N., Askenazi, D., Goldstein, S.L., Fredericks, E.M., McDiarmid, S., Williams, L., Limbers, C.A., Tuzinkiewicz, K., Lerret, S., Alonso, E.M., & Varni, J.W. (2010). Pediatric health-related quality of life: Feasibility, reliability and validity of the PedsQL™ Transplant Module.American Journal of Transplantation, 10, 1677-1685.

© Mapi Research Trust. The unauthorized modification and use of any portion of this document is prohibited.

N° de identificação: ____________

Data: ______________________

PedsQL™

Questionário pediátrico sobre qualidade de vida

Versão 4.0 – Português (Brasil)

RELATO DO/A ADOLESCENTE (13 a 18 anos)

INSTRUÇÕES

A próxima página contém uma lista de coisas com as quais você pode ter dificuldade.
Por favor, conte-nos se **você tem tido dificuldade** com cada uma dessas coisas durante o **ÚLTIMO MÊS**, fazendo um "X" no número:

0 se você **nunca** tem dificuldade com isso
1 se você **quase nunca** tem dificuldade com isso
2 se você **algumas vezes** tem dificuldade com isso
3 se você **muitas vezes** tem dificuldade com isso
4 se você **quase sempre** tem dificuldade com isso

Não existem respostas certas ou erradas.
Caso você não entenda alguma pergunta, por favor, peça ajuda.

Copyright © 1998 JW Varni, Ph.D. Todos os direitos reservados.
Não pode ser reproduzido sem autorização prévia.

Durante o ÚLTIMO MÊS, você tem tido ***dificuldade*** *com cada uma das coisas abaixo?*

**SOBRE MINHA SAÚDE E MINHAS ATIVIDADES** *(dificuldade para...)*	Nunca	Quase nunca	Algumas vezes	Muitas vezes	Quase sempre
1. Para mim é difícil andar mais de um quarteirão	0	1	2	3	4
2. Para mim é difícil correr	0	1	2	3	4
3. Para mim é difícil praticar esportes ou fazer exercícios físicos	0	1	2	3	4
4. Para mim é difícil levantar coisas pesadas	0	1	2	3	4
5. Para mim é difícil tomar banho de banheira ou de chuveiro sozinho/a	0	1	2	3	4
6. Para mim é difícil ajudar nas tarefas domésticas	0	1	2	3	4
7. Eu sinto dor	0	1	2	3	4
8. Eu tenho pouca energia ou disposição	0	1	2	3	4

**SOBRE MEUS SENTIMENTOS** *(dificuldade para...)*	Nunca	Quase nunca	Algumas vezes	Muitas vezes	Quase sempre
1. Eu sinto medo	0	1	2	3	4
2. Eu me sinto triste	0	1	2	3	4
3. Eu sinto raiva	0	1	2	3	4
4. Eu durmo mal	0	1	2	3	4
5. Eu me preocupo com o que vai acontecer comigo	0	1	2	3	4

**COMO EU CONVIVO COM OUTRAS PESSOAS** *(dificuldades para...)*	Nunca	Quase nunca	Algumas vezes	Muitas vezes	Quase sempre
1. Eu tenho dificuldade para conviver com outros / outras adolescentes	0	1	2	3	4
2. Os outros / as outras adolescentes não querem ser meus amigos / minhas amigas	0	1	2	3	4
3. Os outros / as outras adolescentes implicam comigo	0	1	2	3	4
4. Eu não consigo fazer coisas que outros / outras adolescentes da minha idade fazem	0	1	2	3	4
5. Para mim é difícil acompanhar os / as adolescentes da minha idade	0	1	2	3	4

**SOBRE A ESCOLA** *(dificuldades para...)*	Nunca	Quase nunca	Algumas vezes	Muitas vezes	Quase sempre
1. É difícil prestar atenção na aula	0	1	2	3	4
2. Eu esqueço as coisas	0	1	2	3	4
3. Eu tenho dificuldade para acompanhar a minha turma nas tarefas escolares	0	1	2	3	4
4. Eu falto à aula por não estar me sentindo bem	0	1	2	3	4
5. Eu falto à aula para ir ao médico ou ao hospital	0	1	2	3	4

Copyright © 1998 JW Varni, Ph.D. Todos os direitos reservados.
Não pode ser reproduzido sem autorização prévia.

N° de identificação: ____________

Data: ______________________

PedsQL™

Questionário pediátrico sobre qualidade de vida

Versão 4.0 – Português (Brasil)

RELATO DA CRIANÇA (8 a 12 anos)

INSTRUÇÕES

A próxima página contém uma lista de coisas com as quais você pode ter dificuldade.
Por favor, conte-nos se **você tem tido dificuldade** com cada uma dessas coisas durante o **ÚLTIMO MÊS**, fazendo um "X" no número:

0 se você **nunca** tem dificuldade com isso
1 se você **quase nunca** tem dificuldade com isso
2 se você **algumas vezes** tem dificuldade com isso
3 se você **muitas vezes** tem dificuldade com isso
4 se você **quase sempre** tem dificuldade com isso

Não existem respostas certas ou erradas.
Caso você não entenda alguma pergunta, por favor, peça ajuda.

Copyright © 1998 JW Varni, Ph.D. Todos os direitos reservados.
Não pode ser reproduzido sem autorização prévia.

*Durante o ÚLTIMO MÊS, você tem tido **dificuldade** com cada uma das coisas abaixo?*

**SOBRE MINHA SAÚDE E MINHAS ATIVIDADES** *(dificuldade para...)*	Nunca	Quase nunca	Algumas vezes	Muitas vezes	Quase sempre
1. Para mim é difícil andar mais de um quarteirão	0	1	2	3	4
2. Para mim é difícil correr	0	1	2	3	4
3. Para mim é difícil praticar esportes ou fazer exercícios físicos	0	1	2	3	4
4. Para mim é difícil levantar coisas pesadas	0	1	2	3	4
5. Para mim é difícil tomar banho de banheira ou de chuveiro sozinho/a	0	1	2	3	4
6. Para mim é difícil ajudar nas tarefas domésticas	0	1	2	3	4
7. Eu sinto dor	0	1	2	3	4
8. Eu me sinto cansado/a	0	1	2	3	4

**SOBRE MEUS SENTIMENTOS** *(dificuldade para...)*	Nunca	Quase nunca	Algumas vezes	Muitas vezes	Quase sempre
1. Eu sinto medo	0	1	2	3	4
2. Eu me sinto triste	0	1	2	3	4
3. Eu sinto raiva	0	1	2	3	4
4. Eu durmo mal	0	1	2	3	4
5. Eu me preocupo com o que vai acontecer comigo	0	1	2	3	4

**COMO EU CONVIVO COM OUTRAS PESSOAS** *(dificuldades para...)*	Nunca	Quase nunca	Algumas vezes	Muitas vezes	Quase sempre
1. Eu tenho dificuldade para conviver com outras crianças	0	1	2	3	4
2. As outras crianças não querem ser minhas amigas	0	1	2	3	4
3. As outras crianças implicam comigo	0	1	2	3	4
4. Eu não consigo fazer coisas que outras crianças da minha idade fazem	0	1	2	3	4
5. Para mim é difícil acompanhar a brincadeira com outras crianças	0	1	2	3	4

**SOBRE A ESCOLA** *(dificuldades para...)*	Nunca	Quase nunca	Algumas vezes	Muitas vezes	Quase sempre
1. É difícil prestar atenção na aula	0	1	2	3	4
2. Eu esqueço as coisas	0	1	2	3	4
3. Eu tenho dificuldade para acompanhar a minha turma nas tarefas escolares	0	1	2	3	4
4. Eu falto à aula por não estar me sentindo bem	0	1	2	3	4
5. Eu falto à aula para ir ao médico ou ao hospital	0	1	2	3	4

Copyright © 1998 JW Varni, Ph.D. Todos os direitos reservados.
Não pode ser reproduzido sem autorização prévia.

Supplementary Material S5

Rego et al. Transcutaneous posterior tibial nerve stimulation: an adjuvant treatment for intractable constipation in children

Assessment of Quality of Life in Children and Adolescents with Fecal Incontinence (AQLCAFI).

A. Individual and social questions

Below, we will ask other questions to determine if your bowel function bothers you and how often it bothers you. If this complaint exists but it is not because of poor bowel function, please leave the question blank.

	Almost always	Sometimes	Rarely	Never
1 When I am away from home, I try to stay near the bathroom				
2 I avoid visiting my friends				
3 I am not often invited to parties or to go on trips				
4 I avoid spending the night away from home				
5 I fear that people may smell feces or flatus				
6 I would rather stay home than go out				
7 I avoid eating out				
8 I am unable to participate in activities with my friends				
9 I avoid talking about the problem with others				
10 I need to plan my activities according to my bowel function				
11 It impairs my school performance				
12 It affects my professional work				
13 I release stool without noticing				
14 I prefer for people not to be aware of my problem				
15 It hinders the practice of sports				
16 I avoid traveling				
17 I have a difficult time making friends				
18 I worry about accidents with feces				
19 Leaving home worries me				
20 I stop doing the things I like				
21 I feel that I cannot control my bowel movements				
	Poor	Fair	Good	Excellent
22 In general, you think your health is:				
23 How would you rate your bowel function?				
24 In your case, if it was indicated, would you accept other forms of treatment or surgeries to improve your bowel function?				

- Tannuri AC, Ferreira MA, Mathias AL, Tannuri U. Long-term results of the Duhamel technique are superior to those of the transanal pullthrough: A study of fecal continence and quality of life. J Pediatr Surg. 2017 Mar;52(3):449-453.
- Mathias AL, Tannuri AC, Ferreira MA, Santos MM, Tannuri U. Validation of questionnaires to assess quality of life related to fecal incontinence in children with anorectal malformations and Hirschsprung's disease. Rev Paul Pediatr. 2016 Jan-Mar;34(1):99-105.

Supplementary Material S6

Rego et al. Transcutaneous posterior tibial nerve stimulation: an adjuvant treatment for intractable constipation in children

QUESTIONNAIRE FOR THE EVALUATION OF THE APPLICABILITY OF PTNS DAILY HOME SESSIONS

1. How would you rate your experience with electrical stimulation?
○ Poor ○ Bad ○ Fair ○ Great

2. Do you think it is very difficult to do therapy at home? ○ Yes ○ No

3. What has been the greatest difficulty? ○ electrodes ○ regulation of the device ○ ability to connect ○ Acceptance of child
○ Other, Explain this difficulty in more detail ____

4. Does your child have any pain during the application? ○ Yes ○ No
Explain this pain in greater detail____

5. Local physical examination

Surface Sensitivity: ○ Hypoesthesia ○ Normoesthesia ○ Hyperesthesia
Deep sensitivity: ○ Hypoesthesia ○ Normoesthesia ○ Hyperesthesia
Skin integrity: ○ Normal ○ Altered
Inflammatory signs: ○ Present ○ Absent
